# Evaluation of Ultrasound-Guided Radiofrequency Ablation as a Treatment Option for Papillary Thyroid Microcarcinoma in the Isthmus: A Retrospective Study

**DOI:** 10.3389/fendo.2020.599471

**Published:** 2021-02-09

**Authors:** Qing Song, Hanjing Gao, Xiaoqi Tian, Ling Ren, Yu Lan, Lin Yan, Yukun Luo

**Affiliations:** ^1^ Department of Ultrasound, First Medical Center of General Hospital of Chinese PLA, Beijing, China; ^2^ Department of Ultrasound, Seventh Medical Center, General Hospital of Chinese PLA, Beijing, China; ^3^ Department of Ultrasound, General Hospital of Central Theater Command, Wuhan, China

**Keywords:** papillary thyroid carcinoma, ultrasonography, radiofrequency ablation, isthmus, treatment

## Abstract

**Background:**

About 3–9.2% of papillary thyroid carcinomas (PTC) are found in the isthmus, which has unique anatomic properties, making treatment more challenging. The aim of this study was to evaluate the treatment and undesirable effects of ultrasound-guided radiofrequency ablation (RFA) for PTC in the isthmus.

**Methods:**

This retrospective case series study assessed 112 patients with single papillary thyroid microcarcinoma in the isthmus, pathologically diagnosed before RFA at the General Hospital of Chinese PLA in 2014–2018. Follow-up was performed by contrast-enhanced ultrasound (CEUS) and ultrasound examinations at 1, 3, and 6 months and every 6 months thereafter. The complete ablation (CAR), disappearance (DR), and volume reduction (VRR) rates of nodules, the incidence of complications, and the rate of lymph-node metastasis were recorded.

**Results:**

The CAR of the tumors was 100%. During follow-up, the volume of coagulation necrosis gradually decreased. DRs at 1, 3, 6, 12, and 18 months after RFA were 0.8% (1/112), 10.7% (12/112), 51.7% (58/112), 91.0% (102/112), and 100% (112/112), respectively. The VRR evaluated by ultrasound and CEUS gradually increased. One recurrent case (0.8%) was found at 7 months after RFA. No complications, lymph node metastasis confirmed by ultrasound, and abnormal thyroid function were observed.

**Conclusions:**

This retrospective study shows that RFA is beneficial for the treatment of PTMC in the isthmus.

## Introduction

Papillary thyroid carcinoma (PTC) is the most common pathological type of primary thyroid cancer, accounting for approximately 85% of such cancers and showing an increasing incidence recently (1). PTCs are mostly asymptomatic and well-differentiated, with low malignancy, slow growth, and relatively good patient prognosis (2, 3). Papillary carcinoma measuring 10 mm or less is defined as papillary thyroid microcarcinoma (PTMC) by the World Health Organization (WHO) classification (4).

Recently, the incidence of PTMC has overtly increased thanks to broad screening and technological advances in thyroid ultrasound and fine-needle aspiration biopsy (FNAB) (5). The American Thyroid Association (ATA), British Thyroid Association (BTA), and European Society of Medical Oncology (ESMO) recommend that low-risk PTMCs can be periodically followed, with surgery being not immediately necessary due to the good prognosis (2, 6, 7). Nevertheless, no clinical, imaging, molecular, or other parameters accurately differentiate the few aggressive PTMCs from the large proportion of indolent tumors (8).

In recent years, radiofrequency ablation (RFA) for the treatment of benign thyroid nodules and thyroid carcinoma has attracted increasing attention from doctors and patients as a minimally invasive, easy to operate, safe, and predictable procedure, with few complications and high postoperative quality of life (9–13). Meanwhile, about 3–9.2% PTCs are found in the isthmus, which has unique biological and anatomic characteristics, making treatment more challenging (14). To date, there is no recommended standard therapy for PTC in the isthmus (15). In addition, RFA for PTMC in the isthmus has not been assessed.

Therefore, the present study aimed to evaluate the treatment and undesirable effects of RFA for PTMC in the isthmus. The results revealed a promising value for RFA in the treatment of PTMC in the isthmus.

## Materials and Methods

### Patients

**Figure 1:**
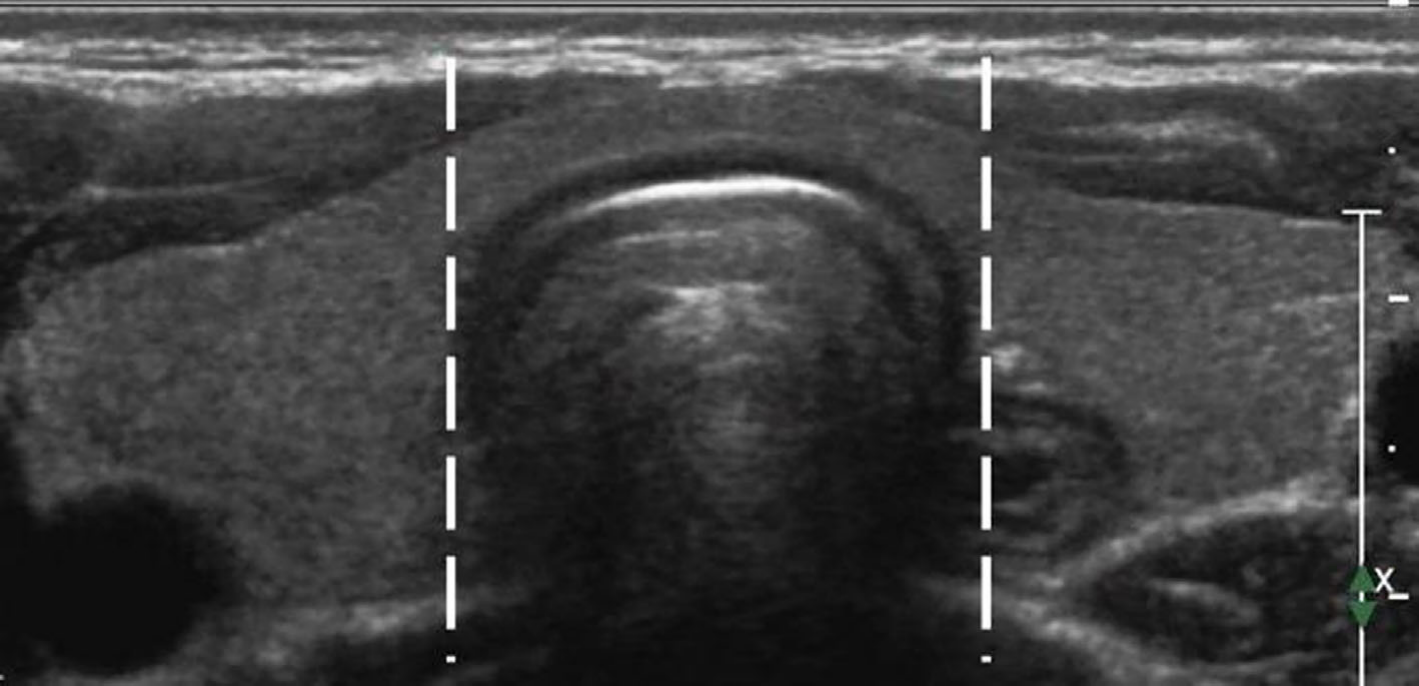
A diagram of the thyroid isthmus.The isthmus is the thyroid tissue between the lateral side-lines of the trachea, delineated by the two dotted lines.

This was a retrospective case series study performed at the General Hospital of the Chinese PLA of patients pathologically diagnosed of PTMC from May 2014 to Apr 2018 by core needle biopsy before RFA. The inclusion criteria were: 1) single nodule smaller than 10 mm; 2) nodule located in the isthmus (**Figure 1**), the capsule/envelope around the nodule was smooth and continuous, there were cross-movements between the thyroid muscle and anterior cervical muscle group, and no signs of invasion of the anterior cervical muscles when swallowing; 3) after adequate communication with the patients, the patients agreed and underwent RFA. The exclusion criteria were: (1) incomplete data; or (2) unclear image. This work has been carried out in accordance with the Declaration of Helsinki (2000) of the World Medical Association. The study was approved by the Ethics Committee of PLA General Hospital (S2019-211-01). There was no requirement for informed consent because of the retrospective design, but all patients signed a treatment consent form.

### Preoperative Preparation

All patients underwent contrast-enhanced ultrasound (CEUS) and ultrasound examinations before RFA. The diameter, volume (V = length × width × depth × 0.524), location, echo property, calcification, and relationship with the capsule membrane of each nodule were evaluated by ultrasound. The blood supply in the nodule was detected by CEUS. CEUS and ultrasound were performed independently by two skillful sonographers with over 10 years of work experience. Then, two doctors with over 5 years of experience analyzed all video data separately. Blood tests, coagulation function evaluation, thyroid hormone level assessment, and electrocardiography (ECG) were performed. The patients only underwent local infiltration anesthesia in the interventional ultrasound room.

### RFA

A specific operation room for all RFA procedures was allocated in the outpatient department. The patients were treated by an experienced sonographer with a work experience of over 10 years, using the Celon AG RFA System (Olympus, Tokyo, Japan) with a disposable bipolar electrode (18G, Olympus). The electrode was a straight tip, with a 9-mm long active component and 3 to 6 W of power. An Acuson Sequoia 512 ultrasound diagnostic system equipped with an L3-6 high-frequency Linear Array Probe (3–6 MHz) was used as a monitoring device during the procedure. The devices for perioperative examination and follow-up included an Acuson Sequoia 512 ultrasound diagnostic system equipped with an L8-15 high-frequency linear array probe (8-15 MHz), and an IU22 Doppler ultrasonic diagnosis apparatus with an L12-5 high-frequency Linear Array Probe (Philips, Best, The Netherlands). The contrast agent used in CEUS was SonoVue (Bracco S.p.A Inc., Milan, Italy); 59 mg of SonoVue was dissolved with 0.9% sodium chloride solution to form a microbubble suspension in a total of 5 ml. After shaking, the contrast agent was injected rapidly through the antecubital vein. Instant monitoring of blood pressure, oxygen saturation, heart rate, and ECG was carried out throughout the RFA process.

The patient lay in the supine positions with the neck in overextension, providing a comfortable posture throughout the procedure. Then, the operation field was conventionally disinfected and covered with sterile surgical towels. Local anesthesia with 1% lidocaine was performed at the puncture site. Before ablation, the hydrodissection technique was performed with a 23-gauge needle to inject sterile saline containing 0.0005% adrenaline between the isthmus and the anterior cervical soft tissue as well as between the isthmus and the trachea to form an isolation zone of at least 1 cm wide to prevent thermal injury.

**Figure 2:**
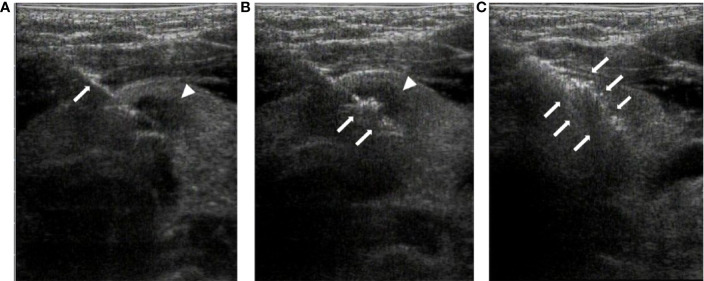
Radiofrequency ablation of thyroid isthmus nodules under ultrasound guidance. **(A)**. The radiofrequency needle (arrow) is advanced into the posterior of the tumor (arrowhead) under ultrasound guidance. **(B)**. At the beginning of RFA, a vaporized area (arrow) appears around the point of the needle, with the tumor still visible (arrowhead). **(C)**. At the end of the ablation, the tumor is completely covered by a transient hyperechoic zone (arrow).

The direct nodule puncture approach was used, and the electrode was advanced into the deepest part of the nodule under ultrasound guidance. Once the electrode was appropriately positioned, the RFA applicator was initiated with an output power of 3–6 W. The “pull-back” technique was applied during the procedure. At the very beginning of RFA, a vaporized area usually appears around the electrode tip, and RFA was performed until the nodule was completely covered by a transient hyperechoic zone, indicating the termination of RFA. The ablated area was evaluated by CEUS, and no contrast perfusion into the ablated area was defined as complete ablation (16). Otherwise, immediate supplementary RFA was recommended to eliminate the residual tumor. **Figure 2** summarizes the procedure.

### Postoperative Evaluation and Follow-Up

After the surgery, the patients were monitored for vital signs and procedure-related adverse events in the operation room for 1–2 h. Follow-up was performed at 1, 3, and 6 months and every 6 months thereafter by ultrasound (volume of the ablated area and lymph-node metastasis in the neck) and CEUS (volume of coagulation necrosis area and remnant or recurrence of tumor).

No contrast perfusion into the ablated area was defined as complete ablation. If the tumor were larger than previously assessed, the presence of surviving tumor cells could not be ruled out, and another RFA was recommended to eliminate the residual tumor. When the tumor became smaller or in case of complete absorption, ablation was then not required. Moreover, a hard-to-evaluate linear scar in the ablation zone was considered to indicate complete absorption.

The complete ablation rates (CAR), disappearance rates (DR), and volume reduction rates (VRR) for nodules (17) were derived at each follow-up point. CAR refers to the coagulative necrosis region caused by ablation totally covered the tumor region, as confirmed by CEUS immediately evaluated at the end of RFA. The CAR is used to determine whether an immediate second ablation is necessary. The DR refers to the speed of the total absorption/disappearance of the coagulative necrosis region caused by ablation during follow-up.

### Statistical Analysis

All data were analyzed using SPSS 25.0 (IBM, Armonk, NY, USA). Continuous variables with a normal distribution are presented as means ± standard deviations and were analyzed using the paired samples t-test. Categorical data are presented as percentages and frequencies. P<0.05 was considered statistically significant.

## Results

### Patient Baseline Features

A total of 163 patients who underwent RFA ablation on isthmus MPTCs were eligible according to the inclusion criteria, and 46 and 5 patients among them were excluded due to incomplete data and unclear image respectively. Eventually, 112 patients with complete follow-up data were included. They were 44.9 ± 10.6 years old, ranging from 22 to 66 years. Among them, 18 patients were male, and 94 were female. The average follow-up duration was 30.2 ± 13.9 months (13–60 months). The average nodule diameter in the 112 cases was 6.5 ± 1.9 mm, for an average volume of 181.6 mm^3^. All of the nodules in the 112 patients showed hypoecho on sonography. No extra-thyroid invasion were detected by sonography in any of the patients. Ten (8.9%) were presented with bulky calcification; 2 (1.8%) showed surrounding annular calcifications; and 34 (30.4%) were presented with punctate calcification; while 66 (58.9%) did not show calcification. Twenty-two (19.6%) of the 112 patients had diffuse lesion. Seventy (62.5%) patients had nodules of aspect ratio less than one, while 42 (37.5%) patients had nodules of aspect ratio larger than one.

One patient was not a surgical candidate (involuntary twitch and tic and renal failure managed with regular hemodialysis), and the other patients were unwilling to undergo surgery and have to take lifelong medication, and surgery was kept in case of tumor progression, recurrence, or relapse. The average RFA energy was 0.78 ± 0.73 kJ (range, 0.39–1.67).

### Clinical Outcomes

There was one recurrent case (0.8%) at 7 months after RFA. No complications, lymph node metastasis confirmed by ultrasound, or abnormal thyroid functions were detected in the study cohort.

**Figure 3:**
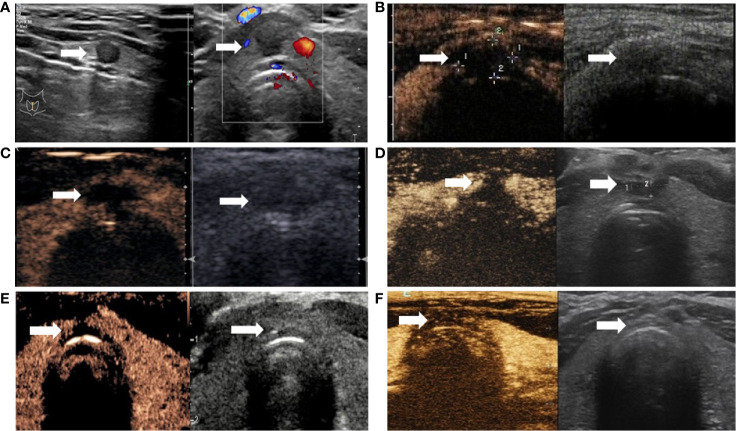
Changes of PTMC in the isthmus after ablation. **(A)** Ultrasound revealing hypoechoic nodules in the isthmus (arrow); left and right panels are sagittal and axial images, respectively. **(B)** Immediately after radiofrequency ablation, CEUS was performed (left and right panels are double-amplitude mode-CEUS and conventional ultrasound images, respectively); no enhancement is observed in the coagulation necrosis area (arrow) as a result of RFA. **(C–F)**. CEUS and conventional ultrasonography images at 1, 3, 6, and 12 months after RFA, respectively. With time, the volume of the coagulation necrosis area (arrow) under CEUS and conventional ultrasonography gradually decreases until the necrotic area is completely absorbed at 12 months.

**Figure 4:**
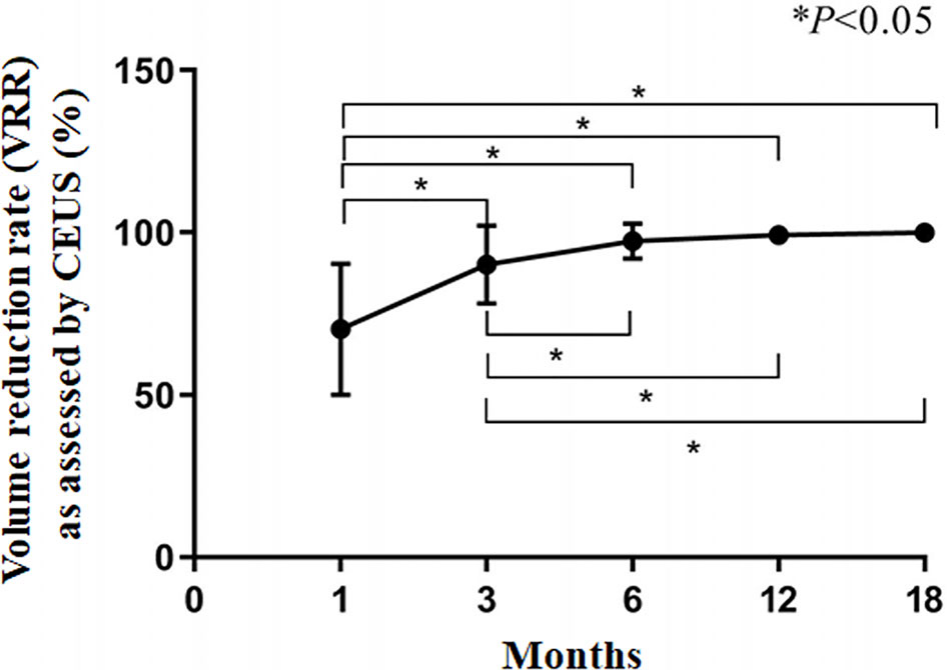
Volume reduction rate (VRR) as assessed by CEUS. *P<0.05. The results are presented as the means ± standard deviations of all patients.

**Figure 5:**
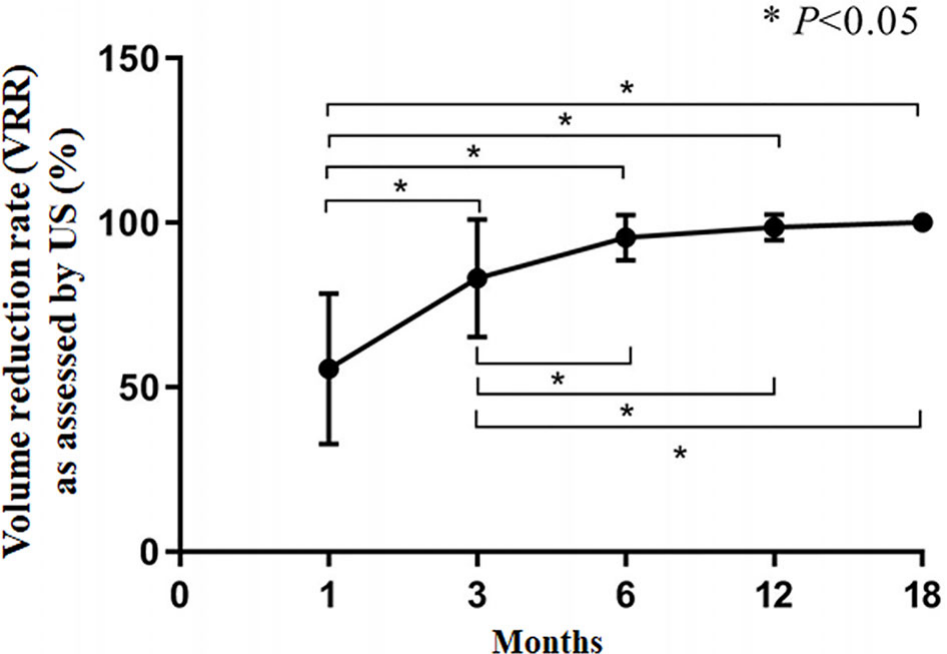
Volume reduction rate (VRR) as assessed by US. *P<0.05. The results are presented as the means ± standard deviations of all patients.

The CAR of the tumors was 100%. During follow-up, the volume of coagulation necrosis gradually decreased (**Figure 3**). The DRs of the nodules at 1, 3, 6, 12, and 18 months after RFA were 0.8% (1/112), 10.7% (12/112), 51.7% (58/112), 91.0% (102/112), and 100% (112/112), respectively. The VRR after RFA, evaluated by US and CEUS, increased with time. VRRs were compared pairwise after treatment, and a significantly higher value was found at 3 months compared with 1 month (*P*<0.001), while there were no significant differences for the other comparisons (P>0.05; **Figures 3**–**5**). Moreover, the ablation zone in one patient was confirmed as total absorption at only 1 month after RFA.

## Discussion

This retrospective study strongly suggests that RFA is beneficial for the treatment of PTMC in the isthmus, reaching the maximum VRR within 6 months after surgery.

The incidence of differentiated thyroid carcinoma (DTC) in the isthmus ranges from 1% to 9.2% (18–21). Treatment of PTC in either lobe has clear recommendations, but the best option for treating PTC in the isthmus remains controversial, as highlighted by the ATA and British guidelines (2, 6, 22). For patients with stage T1b and T2 PTC (no lymph node metastasis), the revised version of the ATA guidelines recommends lobectomy plus isthmusectomy rather than the previous recommendation (2009) of total thyroidectomy (2, 23). The alteration in guidelines reflects that conservative surgical approaches are preferred by clinicians versus extensive radical operations, partially because the latter might induce multiple complications such as hypoparathyroidism and recurrent laryngeal nerve injury, reducing the quality of life (24, 25). Total thyroidectomy is recommended for patients diagnosed with lymph node metastasis of thyroid carcinoma in the 2015 ATA guidelines. Nevertheless, in patients whose malignant lesions are restricted in the isthmus with no lymph node metastasis, neither the extent of operation nor a clear therapeutic strategy is found in the ATA or BTA guidelines (2, 22).

Studies assessing PTC restricted to the thyroid isthmus have indicated that isthmusectomy or limited neck lymph node dissection (lymph nodes anterior to the cricoid cartilage and trachea) shows satisfactory therapeutic effects (24, 26, 27). Specifically, in patients with PTC restricted to the isthmus, especially those with a maximum diameter of less than 10 mm, isthmusectomy or wide-field isthmusectomy (resection of the isthmus and the neighboring lobe of the thyroid) might be a suitable choice because of reduced odds of injuries to the recurrent laryngeal nerve and parathyroid glands. Averagely, the above studies lasted 124 months, and the 10-year disease-specific survival rate and the 10-year negative local recurrence rate were both 100%.

Small-volume PTMC might not lead to poor prognosis with positive monitoring. Indeed, Ito et al. (28) and Ode et al. (27) indicated that after 10 years of follow-up of patients with minor PTC, the mortality rates were similar in the positive monitoring and immediate operation groups. Undoubtedly, patients undergoing positive monitoring should be provided with adequate psychological counseling and management.

Thermal ablation is extensively applied for the treatment of thyroid nodules, PTC, and recurrent thyroid carcinoma with minimal invasion (11, 12, 29–33). A statement from Italy highlights that RFA will play an important role in the future (13), while a recent meta-analysis indicates that RFA is effective and safe in non-surgical candidates (34). It is usually performed by the trans-isthmic approach and moving shot technique. Monopole radiofrequency ablation (RFA) is currently the most documented thermo-ablative method. RFA produces coagulative necrosis within the nodule, and normal thyroid tissue can be preserved as much as possible to ensure the safety of the adjacent membrane and vital organic tissue. After RFA, the necrotic tissue is gradually absorbed, and the symptoms caused by the nodule are relieved (11, 35). A previous study by our team indicated that RFA is very effective in treating low-risk PTC with a relatively low incidence of complications (11), similar to other findings (36). Specifically, 41.7% of nodules were absorbed in 6 months, and this ratio rose to 95.8% at 12 months. These studies revealed RFA to be minimally invasive, easy to operate, safe, predictable, simple, and efficient, which, in turn, attenuates patient anxiety and improves the quality of life.

Nevertheless, the role of RFA in the treatment of PTC in the isthmus remains unclear. In this study, all the nodules were successfully ablated at the first attempt, and no severe complications and thyroid dysfunction were detected. We advanced the probe from one side of the neck, and the anterior and posterior capsules of the isthmus were ablated simultaneously due to the thin anatomic structure of the isthmus. Some patients might feel a dull pain in the neck to some extent, but such discomfort usually disappeared within 1 month. All patients underwent CEUS and ultrasound examination during follow-up, and nodule volumes decreased with time: VRRs were 97.6 ± 5.2% at 6 months and 99.6 ± 1.8% at 12 months; at 18 months, all nodules had disappeared. In one patient, the nodule disappeared at 1 month after RFA, which might be related to the abundant blood supply of the isthmus (37). Normally, a scar may be left after nodule absorption. There was one recurrent case [recurrence >6 months after RFA (38)] in which the primary nodule had multiple microcalcifications, possibly suggesting that RFA might not be suitable for this type of lesion, but this will have to be verified in the future studies.

This study has several limitations. First, it was a retrospective study, with all the inherent drawbacks. In addition, the sample size was relatively small, and the study was performed at a single center. Finally, the follow-up was only 30.2 ± 13.9 months. Therefore, further well-designed, large, multicenter studies are warranted to confirm the current findings and advocate for the wide application of RFA for PTMC therapy.

In summary, this preliminary study showed that RFA is effective in the treatment of thyroid carcinoma in the isthmus, with virtually no complications. However, these findings should be confirmed in future studies.

## Data Availability Statement

The raw data supporting the conclusions of this article will be made available by the authors, without undue reservation.

## Ethics Statement

The studies involving human participants have been carried out in accordance with the Declaration of Helsinki (2000) of the World Medical Association. The study was approved by the Ethics Committee of PLA General Hospital (S2019-211-01). There was no requirement for informed consent because of the retrospective design. Written informed consent for participation was not required for this study in accordance with the national legislation and the institutional requirements.

## Author Contributions

QS, HG, and XT carried out the studies, participated in data collection, and drafted the manuscript. LR, YL, and LY performed the statistical analysis and critical revision for important intellectual content. YKL participated in the acquisition, analysis, and interpretation of data and drafted the manuscript. All authors contributed to the article and approved the submitted version.

## Funding

This project funded by China Postdoctoral Science Foundation (2018M643876) and Innovation Cultivation Fund Project of PLA Army General Hospital (2015-LC-05).

## Conflict of Interest

The authors declare that the research was conducted in the absence of any commercial or financial relationships that could be construed as a potential conflict of interest.
